# A study of docetaxel weekly or every three weeks in combination with carboplatin as first line chemotherapy in epithelial ovarian cancer: Hematological and non-hematological toxicity profiles

**DOI:** 10.3892/ol.2013.1146

**Published:** 2013-01-22

**Authors:** BENGT SORBE, MARIANNE GRAFLUND, LISA NYGREN, GYÖRGY HORVATH

**Affiliations:** 1 Department of Oncology, University Hospital, Örebro;; 2 Department of Oncology, Sahlgrenska University Hospital, Gothenburg, Sweden

**Keywords:** ovarian cancer, docetaxel, carboplatin, weekly administration, 3-week administration, hematological toxicity, non-hematological toxicity

## Abstract

The purpose of this study was to compare the toxicity profiles of docetaxel administered on a weekly schedule and the standard three-week schedule in the treatment of advanced primary ovarian carcinoma. Eligible patients were treated with intravenous docetaxel (30 mg/m^2^) on days 1, 8 and 15, and carboplatin (AUC 5) on day 1 or with docetaxel (75 mg/m^2^) and carboplatin (AUC 5) on day 1; Q21 days for 6 cycles. This study was a pooled study of two primary phase II studies. A total of 108 patients received the weekly schedule and 59 patients received the three-week schedule. All patients were evaluated for toxicity. The overall response rate was 79% and the biochemical response 93% for the weekly schedule. The median overall survival rate was 35.3 months. Neutropenia was significantly more common (ANOVA; p<0.0001) in the three-week group than in the weekly group during all six courses of chemotherapy. Fever and infections were also more common in this group. Thrombocytopenia and anemia were slightly more common in the weekly group. Fatigue, epiphora, nail changes and taste disturbances were specific side-effects following weekly docetaxel. Peripheral sensory neuropathy (grade 1–2) increased with every cycle of treatment, but in a similar manner in the two groups. Grade 3–4 neuropathy was not recorded. Oral mucositis and myalgia were two side-effects associated with the three-week schedule. Nausea and vomiting, diarrhea and dyspnea were a limited problem in both groups. Cardiac toxicity was rare and did not differ between the two docetaxel schedules. The weekly administration was favored due to the lower rates of neutropenia, fever, infections, oral mucositis and myalgia. However, epiphora and nail changes were specific side-effects of the weekly treatment. Both regimens appeared to be rather well tolerated with similar compliance (66 and 70%) with regard to completion of the planned six courses of chemotherapy.

## Introduction

Cancer of the ovary is the seventh most common type of cancer in females, accounting for almost 225,000 new cases and 140,000 mortalities annually (1). Efficacy of first-line chemotherapy with paclitaxel and carboplatin is impressive; however, 70% of patients will eventually succumb to disease-related complications during long-term follow-up.

New chemotherapy agents and alternative dosing schedules have been investigated to improve responses and survival rates and to increase tolerability (2–5). Standard 3-week dosing schedules have improved response rates and progression-free survival, but long-term overall survival rates are less impressive and relapses continue to exceed 70% (3).

Studies have analyzed the efficacy of primary therapy, but data have not shown the superiority of a specific standard triplet chemotherapy regimen for the treatment of ovarian carcinoma (3).

Weekly chemotherapy regimens have been evaluated with regard to improved prognosis and reduced or altered drug toxicity. Promising activity and a favorable toxicity profile have been reported (4,5). A higher total dose of paclitaxel (dose-dense) may be achieved with weekly regimens and may theoretically be superior to the standard 3-week schedule for first-line therapy (6).

Docetaxel is an alternative to paclitaxel in combination with a platinum agent in first-line chemotherapy, but also for the treatment of recurrent ovarian cancer (7,8). The standard three-week schedule appears to be of comparable efficacy with paclitaxel but with a different toxicity profile (9). More dose-dense weekly schedules have also been studied in small patient series of recurrent ovarian cancer (10–16). New data on up-front weekly docetaxel with regard to efficacy, toxicity and quality of life have recently been presented (17,18).

The purpose of the present study was to compare the toxicity profiles of weekly docetaxel administration and the standard three-week schedule for primary therapy of advanced ovarian carcinoma. In both schedules carboplatin was administered every three weeks. Efficacy and quality-of-life data have been presented previously and are not analyzed in this study (17,18).

## Patients and methods

### 

#### Eligibility

This was a retrospective comparative multicenter study, including patients with histologically confirmed epithelial ovarian carcinoma from the Gynecological Oncology departments at two university hospitals (Örebro and Gothenburg) in Sweden. All patients were in FIGO stage IIB–IV and underwent primary cytoreductive surgery. The period of recruitment was from May 2003 to December 2008. In all, 167 patients were included in the study and 108 patients received weekly docetaxel (Örebro) and 59 received docetaxel every three weeks (Gothenburg) together with carboplatin given every three weeks in both regimens. Of these patients, 147 (88%) completed 3 or more courses of chemotherapy. Clinical and biochemical response rates were based on patients with measurable disease and/or elevated CA-125 levels at the start of chemotherapy. The results from a phase II study of weekly treatment have been published previously (17). Toxicity, which is the main topic of this study, was recorded in all 167 patients (≥1 course of chemotherapy). Patient and tumor characteristics are shown in Table I.

A chemotherapy regimen of weekly docetaxel 30 mg/m^2^ and carboplatin [area under the curve (AUC) 5] was given every 3 weeks to 108 patients (17). Six cycles were administered during 18 weeks. Of these patients, 71 (66%) completed 6 cycles of chemotherapy. The mean dose intensity of docetaxel was 29.8 mg/m^2^/week (95% CI, 29.6–29.9) and of carboplatin 105.7 mg/m^2^/week (95% CI, 102.0–109.5).

A chemotherapy regimen of docetaxel 75 mg/m^2^ and carboplatin (AUC 5) was given every 3 weeks to 59 patients (9). Six cycles were administered during 18 weeks. Of these patients, 41 (70%) completed 6 cycles of chemotherapy. The mean dose intensity of docetaxel was 25.1 mg/m^2^/week (95% CI, 24.9–25.3) and of carboplatin 109.4 mg/m^2^/week (95% CI, 105.5–113.3).

Eligible patients had adequate bone marrow, renal and hepatic function, and an absolute neutrophil count (ANC) ≥1.5×10^9^/l, a platelet count ≥100×10^9^/l, serum-creatinine ≤1.25 times the normal level, serum ASAT/ALAT ≤1.5 times the normal level, no previous history of chemotherapy or radiotherapy, and an Eastern Cooperative Oncology Group (ECOG) performance status ≤2. Exclusion criteria included severe infection, hypertension and myocardial infarction within the previous 6 months, congestive heart failure, prior serious allergic reactions and previous malignancy within 5 years. The study was approved by the Ethics Committees of the participating University Hospitals (Dnr 03-258). Informed consent was obtained from all patients.

#### Drug administration

Patients were treated with intravenous docetaxel (30 mg/m^2^) and carboplatin (AUC 5) on day 1. Docetaxel was repeated on days 8 and 15 and was administered via a ½-hour infusion. Carboplatin was administered in accordance with the Calvert formula (19) for 30 min on day 1. In the group with standard chemotherapy, intravenous docetaxel (75 mg/m^2^) and carboplatin (AUC 5) were both given on day 1. The second course started on day 22. Before docetaxel infusion, patients were premedicated with intravenous dexamethasone, diphenhydramine and a histamine H_2_-receptor antagonist, such as cimetidine. The creatinine clearance was calculated by the method of Cockcroft and Gault (20).

#### Response evaluation

Clinical response was assessed at the completion of 6 chemotherapy cycles (or after at least 3 completed cycles) via clinical, radiographic and serologic means in accordance with the WHO response criteria (21) and the Rustin criteria (22). Patients with residual disease at the start of chemotherapy and who completed at least 3 cycles of chemotherapy (n=85) were evaluable for clinical response evaluation. Efficacy data of weekly therapy have been presented in an earlier report (17). In the present study efficacy data were not further analyzed.

#### Toxicity analysis

Toxicity was graded according to the Common Terminology Criteria for Adverse Events (CTCAE v3.0, 2003) (23). Patients were required to have an ANC ≥1.5×10^9^/l and a platelet count ≥100×10^9^/l on day 1 to receive chemotherapy. Complete blood cell values were obtained weekly until the conclusion of cycle 6 and then subsequently, every 3 weeks. Adequate renal function was defined as serum creatinine <1.25 times the upper normal limit, and liver function of bilirubin < upper normal limit, AST/ALT <1.5 times the upper normal limit, and ALP <3 times the upper normal limit. Symptomatic peripheral neuropathy ≥ CTCAE grade 2 was an exclusion criterion. All subjects who completed at least 1 cycle of chemotherapy were included in the toxicity analysis.

A quality-of-life measurement questionnaire (EORTC QOL-C30, version 3) was used in the evaluation of the symptoms recorded during the six courses of treatment (24). The compliance rate was high and 93–99% of the patients had evaluable data. The results from the weekly schedule were analyzed and presented in a prior publication (18).

The median follow-up time of all patients alive was 33 months (range, 1–60 months).

#### Statistical analysis

According to the inclusion and exclusion criteria, 108 patients were included in the one-week group and 59 in the three-week group. Originally the patients were recruited into two separate phase II studies during the same period of time. T-test, Pearson’s Chi-square test and Fisher’s exact test were used to compare continuous and non-continuous data. ANOVA (repeated measurements) was used to compare symptom scores during the whole period of treatment (cycles 1–6). A P-value <0.05 was considered to indicate a statistically significant result. STATISTICA software (StatSoft, Inc., Tulsa, OK, USA) version 10.0 was used for all statistical analyses in this study.

## Results

### 

#### Response and survival rates

In the weekly schedule, 38 patients demonstrated a clinical complete response (44.7%), and 29 patients exhibited a partial response (34.1%), resulting in a total clinical response rate of 78.8% (95% CI, 70.1–87.5%). Data from the three-week schedule showed a clinical response rate of 88.7% (95% CI, 80.2–97.2%).

In the weekly schedule the median overall survival time was 35.3 months and the progression-free survival time was 12.0 months. The corresponding figures in the three-week schedule were 54.1 and 20.0 months, respectively.

Since this was not a randomized study, response and survival data could not be compared compared between the weekly and the three-week schedule due to differences in the study populations; this was not the purpose of the study.

### Toxicity

#### Hematological toxicity

Grade 3-4 neutropenia was recorded in 8 patients (11.3%) in the one-week group and in 32 patients (78.1%) in the three-week group after six completed courses of chemotherapy. This was a highly significant difference (Table II). Grade 1–2 neutropenia was recorded in 29 patients (40.9%) and 6 patients (14.6%) in the two groups, respectively.

**Figure 1:**
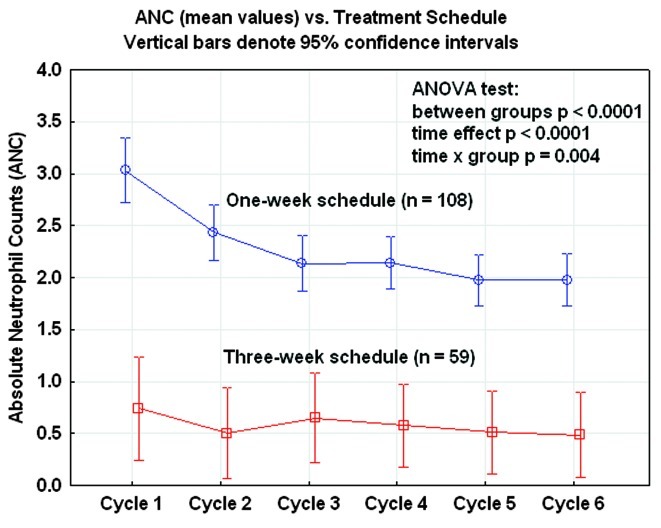
Absolute neutrophil counts (ANC; mean values ± 95% confidence intervals) versus treatment schedule of docetaxel from cycle 1 to cycle 6. Highly significantly differences were noted between the treatments.

During all six courses of chemotherapy the mean neutrophil count was significantly lower in the three-week group compared with the one-week group (ANOVA; p<0.0001). A time-dependent effect was also observed in the one-week group with successively decreasing mean values from cycle 1 to cycle 5. This time-dependent pattern was not observed in the three-week group, with low but stable mean values from cycles 1–6 (Fig. 1).

Febrile neutropenia and septicemia were also more frequent in the three-week group (Table III).

Grade 3–4 thrombocytopenia was recorded in one patient in the one-week group and in no patients in the three-week group. However, grade 1–2 thrombocytopenia was more frequent in the one-week group (50.7%) than in the three-week group (14.6%). The thrombocytic toxicity was more pronounced in the one-week-group and significantly increased with every chemotherapy cycle administered. In the three-week group this pattern was not observed.

None of the patients exhibited grade 3–4 anemia. Grade 1–2 anemia was more frequent in the one-week group (95.8%) than in the three-week group (73.2%). The degree of anemia increased with every successive course of chemotherapy and in a similar manner for both treatment groups. All of these differences were statistically significant (Table II). Despite the different pattern of hematological toxicity, the compliance rate with the chemotherapy regimens was similar in the two groups, and 65.5 and 69.5% of the patients, respectively, completed the planned 6 cycles.

#### Non-hematological toxicity

Fatigue was the most frequently recorded non-hematological side-effect associated with the one-week regimen and was significantly more frequent than with the three-week regimen (ANOVA; p<0.0001). This difference was noted for every individual cycle of the weekly schedule and the time-effect was also present with increasing fatigue during the treatment period (Table IV).

The second most common adverse event was watery eyes and tearing (epiphora), affecting 55 patients (50.9%) in the one-week group but only one patient (1.7%) in the three-week group. Thus, this side-effect was very specific for the weekly regimen and the frequency increased for every consecutive treatment cycle (Fig. 3).

Nail changes were relatively common with the weekly schedule and were reported in 30 cases (27.8%) compared with 9 cases (15.3%) with the three-week schedule (Fisher’s exact test; p= 0.049). Analyzed with ANOVA repeated test, significant differences (p<0.005) were shown after 3 courses of chemotherapy and until 6 completed courses as well as for the complete treatment (course 1–6). A significant time-dependent effect was noted after 3 cycles in the one-week group and after 5 cycles in the three-week group (p<0.0001).

With regard to peripheral neurotoxicity, no significant differences (ANOVA; p=0.125) were reported between the two treatment schedules, but a very pronounced time-effect was observed in both groups (ANOVA; p<0.0001; Table IV). After 6 completed courses of chemotherapy, 28 patients (39.4%) had grade 1–2 neurotoxicity in the weekly group and 13 patients (24.5%) in the three-week group. No patients exhibited grade 3 or higher sensory neuropathy (Table III).

Oral mucositis was significantly more common with the three-week schedule (ANOVA; p<0.0001) and increased in frequency from cycle 1 to cycle 3 and then reached a plateau and slightly decreased up to cycle 6. In the one-week group this was a less common problem but showed a slight increase with every consecutive cycle of treatment.

Taste disturbances were significantly more common after weekly treatment (ANOVA; p<0.0001) and showed a clear cut increase in frequency from the first to the fourth docetaxel cycle and then reached a plateau. In the three-week group this was a minor problem with a different time pattern (Table IV).

Myalgia following treatment was more pronounced with the three-week schedule than with weekly administration of docetaxel. The difference was highly significant (ANOVA; p<0.0001) during the complete period of treatment. There was no significant change in myalgia with time and this was true for both treatment groups (ANOVA; p=0.626).

Cardiac toxicity was extremely rare in the two groups. No significant differences were noted during the treatment period of six administered cycles of docetaxel-carboplatin (ANOVA; p=0.809). No significant time-dependent effect was noted in either group (ANOVA; p=0.210).

The mean score of nausea and vomiting was 14–15 on the scale of 1–100 in both groups after the first course of chemotherapy and then significantly decreased to ∼6 after five courses of therapy (ANOVA; p<0.0001). There were no significant differences between the two chemotherapy regimens (ANOVA; p= 0.277). Dyspnea and diarrhea were slightly more common after weekly administration of docetaxel but of limited clinical significance.

**Figure 2:**
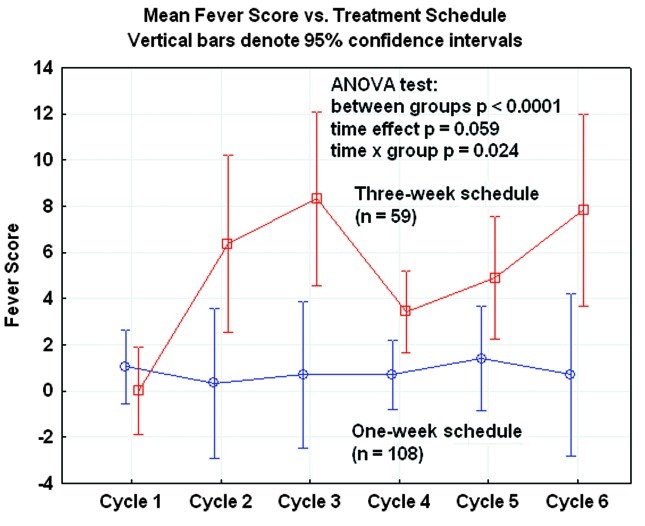
Fever score (mean values ± 95% confidence intervals) versus treatment schedule of docetaxel from cycle 1 to cycle 6. Highly significantly differences were noted between the treatment groups.

**Figure 3:**
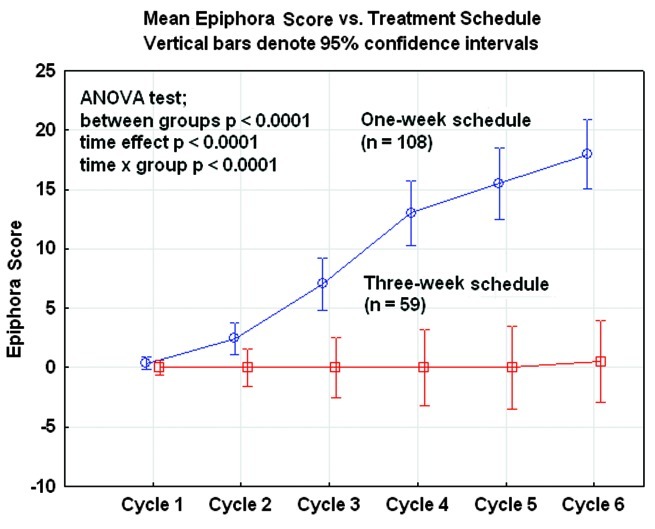
Epiphora score (mean values ± 95% confidence intervals) versus treatment schedule of docetaxel from cycle 1 to cycle 6. Highly significantly differences were noted between the treatments.

Fever and clinical infections were significantly more frequent after three-week administration of docetaxel than after weekly administration (ANOVA; p<0.0001; Fig. 2). This was possibly associated with neutropenia for the three-week schedule.

## Discussion

In order to improve the efficacy and tolerability of standard 3-week regimens of the combination of carboplatin and a taxane, the activity of weekly administration of docetaxel and 3-weekly carboplatin has been studied (17). This has not been performed previously in a first-line setting in primary advanced ovarian cancer. Katsumata *et al*(6) have investigated the efficacy of the combination of weekly dose-dense paclitaxel (80 mg/m^2^) and carboplatin (AUC=6) compared with paclitaxel (180 mg/m^2^) and carboplatin (AUC=6) every 3 weeks in patients with previously untreated ovarian cancer. The two regimens had relatively similar toxicity, but progression-free survival was significantly improved in the patients who received the dose-dense regimen (median 28 versus 17 months; p=0.0014). Weekly chemotherapy data have been reported in a number of previous studies (4,5). In our prior study (17) we encountered an overall response rate of 79%, which is superior to the 56% reported by Katsumata *et al*(8). Our data and results are in line with those of Micha *et al*(25) and Penson *et al*(26) who reported response rates of 80 and 76%, respectively, when adding bevacizumab to paclitaxel and carboplatin in the treatment of advanced-stage ovarian cancer.

The present study focuses on hematological and non-hematological toxicity when docetaxel is administered weekly compared with the standard 3-week schedule in combination with carboplatin given every three weeks. The dose-intensity of docetaxel was higher in the weekly schedule with 29.8 mg/m^2^/week compared to 25.1 mg/m^2^/week in the three-week schedule (t-test; p<0.001). The dose intensity of carboplatin was similar in the two regimens (t-test; p=0.211) with 105.7 and 109.4 mg/m^2^/week, respectively. Thus, differences in toxicity should be associated with the docetaxel treatment schedule. This was not a randomized study, but two separate first-line phase II studies of docetaxel in combination with carboplatin in the primary treatment of advanced ovarian cancer. The two study cohorts were similar with regard to the patients’ characteristics, but with regard to the tumors there were slightly more endometrioid and more stage IIIB tumors in the three-week group, and more papillary serous and stage IV tumors in the one-week group. Distribution of the tumor grade was similar in the two groups. Response rates and survival data for the weekly schedule have been presented in a previous study (17). No comparison was made between the two regimens with regard to response rate and survival in the current study.

In terms of hematological toxicity, grade 3–4 neutropenia was recorded in 11% of the patients after six courses and weekly administration and in 78% after the standard three-week schedule. This was a highly significant difference and it was true during the whole course of chemotherapy from cycle 1 to cycle 6. The higher frequency of fever and infections recorded in the three-week group was probably explained by this myelosuppression. This difference in neutropenia (ANC) was the most important and clinically relevant difference between the two regimens. In a study from Germany, Sehouli *et al*(5) reported that 28% of their patients with advanced ovarian cancer developed grade 3–4 neutropenia after weekly paclitaxel (100 mg/m^2^) and carboplatin (AUC 2). Thrombocytopenia was infrequent and grade 3–4 was recorded in only one patient in the weekly group. However, grade 1–2 thrombocytopenia was more frequent in the one-week group (51%) than in the three-week group (17%), but this difference in laboratory readings had no clinical implications in this series of patients. None of the patients experienced grade 3–4 anemia. Grade 1–2 anemia was recorded in 96% of the weekly group and in 73% of the three-week administration group. This difference was statistically significant (Pearson Chi-square; p<0.0001), but clinically of minor importance. Overall, the hematological toxicity was quite manageable in both groups, but was more so in the weekly schedule with regard to the risk of neutropenia, fever and infections. Hematological toxicity (neutropenia) is also a common reason for not completing all planned courses of a chemotherapy regimen. Colony-stimulating factors were normally not used in these two phase II studies.

In the present study, sensory neuropathy grades 1 and 2 developed in 39.4% of patients in the one-week group and in 24.5% of those in the three-week group after six completed cycles of chemotherapy. Neuropathy significantly increased with time in both groups, but there were no significant differences between the two treatment regimens. None of the patients exhibited severe neurotoxicity grade 3. These results are comparable to those of Micha *et al*(25) and Sehouli *et al*(5) who reported low rates of severe peripheral neuropathy (2.3% grade 3). One would suspect that lower, weekly doses of taxanes would mitigate toxicity (4). However, Seidman *et al*(27) reported that neurotoxicity was a dose-limiting factor after weekly paclitaxel (80 mg/m^2^) for the treatment of metastatic breast cancer. Thus, docetaxel appears to have a lower rate of disabling neurotoxicity than paclitaxel when administered on a weekly schedule. However, the weekly schedule of docetaxel did not appear to be superior to a 3-week schedule of the same drug.

Epiphora was a type of toxicity recorded in 51% of the patients treated with the weekly schedule, but only in 1.7% of the patients with the three-week schedule. This toxicity appears to be specifically associated with weekly administration of docetaxel. Esmaeli *et al*(28) from the MD Anderson Cancer Center reported on 148 patients with this type of side-effect. Thirty of 71 patients given weekly docetaxel needed surgery to correct epiphora. Of the patients who received docetaxel every 2 or 3 weeks, only 3 required a surgical intervention to correct epiphora. A schedule of docetaxel given every 2 weeks has shown a favorable outcome and toxicity profile, also with regard to epiphora, and should be further evaluated in larger series of advanced ovarian cancer patients (29).

Fatigue was the most frequently reported side-effect and was particularly associated with the weekly regimen. The short interval (one week) between the administrations of docetaxel may explain this difference and time for recovery is therefore limited. Nail changes were also a common side-effect of the weekly schedule and were twice as common as in the three-week schedule. This side-effect has also been reported for weekly treatment with paclitaxel and is sometimes quite serious (30).

Oral mucositis may be a problem when higher doses of docetaxel are administered every three weeks (31). This was also confirmed in this study with significantly more mucositis in the three-week group. The incidence of mucositis reached a maximum after the third chemotherapy cycle with docetaxel. On the other hand, taste disturbances were significantly more common after weekly administration than after three-week administration.

Diarrhea and dyspnea were more frequent with the weekly schedule, but these side-effects appeared to be of less clinical significance and few patients reported these symptoms. Cardiac toxicity was also very rare and no differences between the two groups were recorded.

Fever and infections were significantly more frequent in the three-week group and this was a clinically important difference between the two schedules, favoring the weekly schedule. The more pronounced neutropenia after the standard three-week schedule is the probable reason for this difference (32).

In the current study the technique from the quality-of-life analysis (EORTC QLQ-C30) was used to calculate a symptom score on a linear scale from 0 to 100 for each symptom item (33). These scores were also used in the repeated ANOVA analyses when the two treatment schedules were compared over time. In these analyses the differences may be compared between the groups, as well as differences and patterns over time, and the combined effect of group and time.

Quality-of-life measurements were not part of this study, but data from the weekly schedule have been presented previously (18). The results from the quality-of-life data showed similar levels as for the standard carboplatin-paclitaxel regimen administered every three weeks (34). Peripheral neuropathy was one the most notable side-effects affecting quality of life (35). Peripheral neuropathy is a minor problem for docetaxel compared with paclitaxel regimens in the treatment of ovarian cancer. This is the principal advantage of this taxane. One-third of patients undergoing cisplatin and paclitaxel treatment experienced long-term toxicity (36).

The two docetaxel schedules studied showed different toxicity profiles favoring weekly administration with regard to neutropenia, fever and infections as well as problems with oral mucositis and myalgia. Fatigue, epiphora, taste disturbances and nail changes were more specific side-effects of the weekly schedule and in a number of cases a clinical problem. Peripheral sensory neuropathy is a more limited problem with docetaxel compared with paclitaxel but no significant differences were noted between the two regimens studied.

Docetaxel is an alternative to paclitaxel in first-line and second-line chemotherapy regimens for advanced ovarian cancer. Dose-dense schedules with weekly or twice-weekly administrations of the drug should be further explored to improve and optimize the efficacy and the toxicity profile of docetaxel chemotherapy combinations.

## Figures and Tables

**Table I:** Patient characteristics for the series of weekly vs. three-week docetaxel-carboplatin administrations (n=167).

Characteristics	One-week group	Three-week group
Mean age, years	63.3 (range, 28–80)	63.6 (range 47–79)
Body mass index (BMI)	23.8	23.8
Body surface area (BSA), m^2^	1.69	1.67
Histological type, n (%)		
Papillary serous	95 (88.0)	45 (76.3)
Mucinous	2 (1.9)	0 (0.0)
Endometrioid	4 (3.7)	9 (15.3)
Clear cell	7 (6.5)	3 (5.1)
Anaplastic	0 (0.0)	2 (3.4)
FIGO stage, n (%)		
IIB	1 (0.9)	1 (1.7)
IIC	4 (3.7)	4 (6.8)
IIIA	3 (2.8)	2 (3.4)
IIIB	3 (2.8)	8 (13.6)
IIIC	63 (58.3)	31 (52.5)
IV	34 (31.5)	13 (22.0)
Differentiation grade, n (%)		
Poor	67 (62.0)	37 (62.7)
Moderate	29 (26.9)	16 (27.1)
Well	5 (4.6)	3 (5.1)
Not graded (clear cell)	7 (6.5)	3 (5.1)

**Table II:** Hematological toxicity in the one-week and three-week groups.

Toxicity	One-week group	Three-week group	P-value^a^
Neutropenia^b^			
Grade 1	7 (9.9)	1 (2.4)	
Grade 2	22 (31.0)	5 (12.2)	
Grade 3	7 (9.9)	15 (36.6)	
Grade 4	1 (1.4)	17 (41.5)	<0.000001
Thrombocytopenia^b^			
Grade 1	31 (43.7)	5 (9.8)	
Grade 2	5 (7.0)	2 (3.9)	
Grade 3	1 (1.4)	0 (0.0)	
Grade 4	0 (0.0)	0 (0.0)	0.0002
Anemia^b^			
Grade 1	49 (69.0)	18 (35.3)	
Grade 2	19 (26.8)	12 (23.5)	
Grade 3	0 (0)	0 (0.0)	
Grade 4	0 (0)	0 (0.0)	<0.000001

a Pearson Chi-square test;

b Toxicity grading after 6 completed courses of chemotherapy.

**Table III:** Non-hematological toxicity in the one-week and three-week groups.

Type of toxicity^a^	No. of patients (%)				P-value^b^
	Grade 1	Grade 2	Grade 3	Grade 4	
Sensory neuropathy					0.237
One-week group	25 (35.2)	3 (4.2)	0	0	
Three-week group	12 (22.6)	1 (1.9)	0	1 (1.9)	
Nausea					0.626
One-week group	11 (15.5)	2 (2.8)	1 (1.4)	0	
Three-week group	6 (11.8)	3 (5.9)	2 (3.9)	0	
Mucositis					0.403
One-week group	10 (14.1)	1(1.4)	0	0	
Three-week group	4 (7.8)	2 (3.9)	0	0	
Nail changes					0.125
One-week group	13 (18.3)	8 (11.3)	0	0	
Three-week group	8 (15.7)	1 (2.0)	0	0	
Diarrhea					0.033
One-week group	9 (12.7)	0	0	0	
Three-week group	1 (2.0)	0	0	0	
Myalgia					0.163
One-week group	3 (4.2)	0	1 (1.4)	0	
Three-week group	5 (9.8)	2 (3.9)	0	0	
Dyspnea					0.042
One-week group	9 (12.7)	4 (5.6)	1 (1.4)	0	
Three-week group	0	2 (3.9)	0	0	
Cardiac					0.576
One-week group	2 (2.8)	1 (1.4)	1 (1.4)	2 (2.8)	
Three-week group	3 (5.9)	1 (2.0)	0	0	
Fever					0.040
One-week group	2 (2.8)	0	0	0	
Three-week group	0	3 (5.9)	2 (3.9)	1 (2.0)	
Infection					0.824
One-week group	1 (1.4)	3 (4.2)	2 (2.8)	0	
Three-week group	1 (2.0)	4 (8.0)	1 (2.0)	0	
Fatigue					0.496
One-week group	26 (36.1)	7 (9.7)	1 (1.4)	0	
Three-week group	15 (29.4)	3 (5.9)	0	0	
Tearing eyes					0.000
One-week group	37 (52.1)	7 (9.9)	0	0	
Three-week group	1 (2.0)	0	0	0	
Taste disturbances					0.425
One-week group	26 (36.3)	6 (8.5)	0	0	
Three-week group	14 (27.5)	3 (5.9)	0	0	

a Toxicity grading after 6 completed courses of chemotherapy;

b Pearson Chi-square test.

**Table IV:** Non-hematological toxicity in the one-week group and the three-week group during treatment (cycle 1–6). The toxicity grading is converted to a 0–100 linear scale according to the technique used for quality-of-life analysis (EORTC QLQ-C30 symptom scores).

Toxicity	Cycle						P-values (ANOVA)		
	1	2	3	4	5	6	Time	Group	TxG
Neuropathy sensory									
One-week group (A)	2.2	3.2	4.5	7.3	9.0	10.9			
Three-week group (B)	0.0	1.9	2.8	3.8	6.1	8.5			
P-value A vs. B	0.023	0.311	0.274	0.102	0.227	0.402	0.000	0.125	0.971
Fatigue									
One-week group (A)	8.1	11.5	13.7	14.0	15.1	14.9			
Three-week group (B)	0.0	1.4	1.9	1.0	1.9	10.3			
P-value A vs. B	0.000	0.000	0.000	0.000	0.000	0.000	0.000	0.000	0.004
Nausea and vomiting									
One-week group (A)	14.0	12.3	8.6	7.9	9.0	6.3			
Three-week group (B)	15.0	15.6	12.0	9.9	5.3	8.8			
P-value A vs. B	0.750	0.293	0.238	0.428	0.226	0.417	0.000	0.277	0.610
Myalgia									
One-week group (A)	0.7	1.2	1.3	1.5	1.9	2.5			
Three-week group (B)	5.9	6.9	7.4	7.1	7.7	7.4			
P-value A vs. B	0.000	0.000	0.000	0.006	0.014	0.032	0.626	0.000	0.995
Mucositis									
One-week group (A)	2.5	5.4	5.2	4.3	4.2	4.2			
Three-week group (B)	6.5	8.8	13.4	12.7	12.5	9.8			
P-value A vs. B	0.025	0.180	0.001	0.000	0.002	0.013	0.001	0.000	0.106
Taste disturbances									
One-week group (A)	5.6	10.3	13.0	13.4	12.8	13.4			
Three-week group (B)	0.0		1.9	0.9	1.9	1.4	5.9		
P-value A vs. B	0.000	0.000	0.000	0.000	0.000	0.006	0.000	0.000	0.004
Nail changes									
One-week group (A)	0.7	1.7	4.2	4.6	9.6	10.2			
Three-week group (B)	0.0	0.0	0.0	0.0	1.4	4.9			
P-value A vs. B	0.201	0.085	0.005	0.006	0.000	0.001	0.000	0.003	0.012
Dyspnea									
One-week group (A)	1.5	1.5	2.1	3.4	3.6	7.0			
Three-week group (B)	0.0	0.0	0.0	3.3	0.5	2.0			
P-value A vs. B	0.115	0.169	0.079	0.981	0.058	0.046	0.002	0.010	0.113
Cardiac toxicity									
One-week group (A)	0.2	1.5	3.6	3.0	2.6	5.3			
Three-week group (B)	0.5	1.9	2.3	4.7	1.4	2.5			
P-value A vs. B	0.658	0.808	0.577	0.533	0.547	0.342	0.210	0.809	0.399
Epiphora									
P-value A vs. B	0.201	0.013	0.000	0.000	0.000	0.000			
One-week group (A)	0.7	3.7	7.6	12.8	15.1	18.0			
Three-week group (B)	0.0	0.0	0.0	0.0	0.0	0.5	0.000	0.000	0.000
Diarrhea									
One-week group (A)	6.1	7.1	3.4	2.7	3.5	3.2			
Three-week group (B)	1.4	3.8	2.8	1.4	1.5	0.5			
P-value A vs. B	0.016	0.142	0.729	0.334	0.176	0.033	0.023	0.018	0.690
Fever									
One-week group (A)	0.7	0.2	1.3	0.6	1.9	0.7			
Three-week group (B)	0.0	6.5	7.9	3.3	4.8	7.8			
P-value A vs. B	0.465	0.003	0.005	0.014	0.119	0.010	0.059	0.000	0.024
Infection									
One-week group (A)	2.5	1.7	3.9	1.8	1.9	4.6			
Three-week group (B)	0.0	7.4	7.9	4.7	6.3	6.0			
P-value A vs. B	0.117	0.016	0.190	0.208	0.091	0.641	0.058	0.009	0.143
